# Phytochemical Profile, Antioxidant, Anti-Atopic, and Anti-Inflammatory Activities of *Filipendula glaberrima* Nakai at Different Growth Stages

**DOI:** 10.3390/ph17070928

**Published:** 2024-07-11

**Authors:** Hak-Dong Lee, Genevieve Tonog, Neil Patrick Uy, Yunji Lee, Ki-Young Kim, Hangeun Kim, Sanghyun Lee

**Affiliations:** 1 Department of Plant Science and Technology, Chung-Ang University, Anseong 17546, Republic of Korea; gkrehd1234@naver.com (H.-D.L.); thisispatrickuy@gmail.com (N.P.U.); 2 Natural Product Institute of Science and Technology, Anseong 17546, Republic of Korea; 3 Department of Food and Nutrition, Chung-Ang University, Anseong 17546, Republic of Korea; genzkie01@cau.ac.kr; 4 Department of Herbal Crop Research, National Institute of Horticultural and Herbal Science, Eumseong 27709, Republic of Korea; yoong0625@korea.kr; 5 Department of Genetics and Biotechnology, Kyung Hee University, Yongin 17104, Republic of Korea; kiyoung@khu.ac.kr; 6 Research and Development Center, Skin Biotechnology Center Inc., Yongin 17104, Republic of Korea; hkim93@khu.ac.kr

**Keywords:** antioxidant, anti-atopic, anti-inflammatory, *Filipendula glaberrima*, phytochemicals

## Abstract

Since atopic dermatitis is an inflammatory skin disease, natural remedies, such as *Filipendula glaberrima* Nakai (FG), with anti-inflammatory properties are possible promising therapeutic options. This study aimed to investigate the therapeutic potential of FG extracts at different growth stages. Seven compounds were isolated from the FG leaf extracts using open-column chromatography, and they were analyzed using HPLC. The extracts were further evaluated for their total polyphenol and flavonoid content (TPC and TFC). The in vitro antioxidant properties of the FG extracts were evaluated using radical scavenging assays, whereas their anti-inflammatory activities were assessed by evaluating their ability to inhibit the production of inflammation-associated biomarkers using the Griess assay and ELISA, respectively. The MTT assay was used to evaluate the viability and cytotoxicity of the FG extracts in keratinocyte cell lines. The results showed that the full-flowering stage exhibited the highest TPC, TFC, and antioxidant activities, thus suggesting a positive correlation between these properties. All FG extracts showed significant anti-inflammatory activity by inhibiting the production of pro-inflammatory biomarkers in lipopolysaccharide-stimulated macrophages. Additionally, the FG extracts suppressed the production of cytokines and chemokines in keratinocytes, indicating their anti-atopic potential. HPLC analysis revealed that the full-flowering stage had the highest content of all the analyzed phytochemicals (gallic acid, (+)-catechin, hyperin, miquelianin, astragalin, afzelin, and quercetin). These results suggest that the full-flowering stage of FG is the most promising source for therapeutic applications owing to its superior phytochemical profile and biological activities. This study highlights the potential of FG extracts, particularly in its full-flowering stage, as a natural therapeutic agent for the management of inflammation-related diseases, and it can also serve as a reference for further research on FG.

## 1. Introduction

*Filipendula glaberrima* Nakai (FG) is native to South Korea, along with *F. formosa* and *F. koreana* [1]. Most *Filipendula* species (Rosaceae family) are native to Northeast Asia, specifically South Korea, Japan, Manchuria, and Eastern Siberia. The genus *Filipendula*, commonly known as meadowsweets, are perennial herbaceous plants found in temperate regions of the world. These plants are prized for their ornamental value in gardens and parks, with attractive clusters of white or pink flowers and distinctive serrated leaves. However, *Filipendula* is also of great scientific interest for its medicinal properties, particularly as a source of salicylates, which constitute aspirin. Traditionally used to treat fever, headaches, and pain, *Filipendula* has shown potential in the treatment of several conditions including inflammation, arthritis, and cancer [2,3,4].

The isolation of various phytochemicals from *Filipendula* species has been documented, including flavonoids such as rutin, hyperoside, spiraeoside, kaempferol, and (+)-catechin; salicylates such as salicylic acid, spiraein, and methyl salicylate; and tannins, including rugosin D and tellimagrandins I and II [5,6,7,8]. *F. ulmaria*, in particular, has yielded over 40 phytochemicals, with the isolation of a novel flavonoid, ulmarioside. However, the isolations of monotropitin, the rugosin B methyl ester, and the β-sitosterol 3-*O*-β-d-glucoside have been documented in *F*. *glaberrima*, which is a Korean endemic species. Further isolation studies of FG have been pursued [9,10].

Atopic dermatitis, a chronic inflammatory skin disease affecting 10–20% of the population with no definitive cure, is characterized by dysregulated inflammation and immune responses that significantly contribute to its pathogenesis, involving cytokine imbalances and T-cell dysfunction [11]. Although current treatments offer some efficacy, they are often associated with unwanted side effects. Inflammation, mediated by cytokines and inflammatory mediators, such as nitric oxide (NO) and interleukins (ILs), is a hallmark of atopic dermatitis and other chronic diseases [12,13]. Therefore, natural remedies with anti-inflammatory properties hold great promise for the treatment of atopic dermatitis. Consequently, understanding the bioactive compounds present in FG and their antioxidant properties may provide valuable insights into their potential anti-inflammatory and anti-atopic activities. Elucidating the underlying mechanisms of these effects may lead to the development of safer and more effective therapies for atopic dermatitis.

Therefore, we aimed to evaluate the anti-inflammatory and anti-atopic activities of FG leaves extracts obtained from different growth stages in established atopic dermatitis models. In addition to investigating the role of lipopolysaccharide (LPS)-stimulated RAW 264.7 and HaCaT cells, as well as their NO and cytokine production (IL-6, IL-8, monocyte chemoattractant protein-1 [MCP-1], and tumor necrosis factor-alpha [TNF-α]) [14,15], we also aimed to perform a thorough phytochemical analysis of the FG extracts and evaluate their antioxidant activity. These results, together with the evaluation of the effects of FG on immune function, will provide valuable reference data for further research on this understudied species.

## 2. Results and Discussion

### 2.1. Total Polyphenol and Flavonoid Content (TPC and TFC)

In Table 1, the highest TPC, which was determined using gallic acid as a standard, was observed during the full-flowering (FF, 14.75 mg/mL) and 2 months after leaf emergence (LE, 12.75 mg/mL) stages. Conversely, the lowest TPC of 5.53 mg/mL was recorded during the mid-flowering (MF) stage. Similarly, the highest TFC measured with quercetin as the standard was found during the FF (7.75 mg/mL) and LE (7.49 mg/mL) stages. Conversely, the lowest TFC was observed during the pre-flowering (PF, 5.31 mg/mL) and MF (5.37 mg/mL) stages. Our findings are consistent with those of previous studies on *Cleome gynandra* L., also known as African cabbage, which showed the highest accumulation of flavonoids and polyphenols during the flowering stage. Similarly, *Hypericum perforatum*, or Saint John’s wort, had the highest levels of these compounds at the FF stage [16,17,18]. This consistency supports a potential link between the flowering stage and the accumulation of TPC and TFC in plants. Savina et al. [19] suggested that this association may be linked to an increase in the activity of the key enzyme phenylalanine ammonia-lyase, which is involved in phenolic compound synthesis, and potentially alongside increased activity in other enzymes of the flavonoid biosynthesis and transformation pathways. However, our findings contrast with those reported for *Malus* spp. (Rosaceae family), where the TPC and TFC in petals were found to be initially high but then significantly decreased during flower development [20]. These trends, while observed in several Rosaceae plants, can vary based on factors such as plant part, species, cultivar, environmental conditions, and the specific phenolic compounds analyzed.

### 2.2. Antioxidant Activities

The in vitro antioxidant properties of FG extracts were evaluated using 2,2-diphenyl-1-picrylhydrazyl (DPPH) and 2,2′-azino-bis (3-ethylbenzothiazoline-6-sulfonic acid) (ABTS) radical scavenging assays, and the IC_50_ values were compared with those of ascorbic acid, which served as a representative positive control for the antioxidant experiments. Table 1 shows that, while most of the FGs at all developmental stages exhibited significant antioxidant abilities, the extract from the FF stage showed the lowest IC_50_ value in the ABTS (0.16 mg/mL) and DPPH assays (0.55 mg/mL). Compared to the IC_50_ value of 0.1 mg/mL obtained for ascorbic acid, the observed IC_50_ value of FG indicated a remarkably strong antioxidant capacity. Extracts from the LE and early flowering (EF) stages showed the next highest antioxidant capacity in both assays. These trends are consistent with the results of the TPC and TFC experiments, which suggested a positive correlation between the flavonoid and polyphenol contents of FG and their antioxidant capacity.

Ayele et al. reported that the root extract of *Croton macrostachyus* with the highest TPC (1557 mg GAE/100 g) and TFC (745 mg catechin equivalents/100 g) content also showed the strongest antioxidant activity (3.5 mg ascorbic acid equivalents/g) [21]. Another study on avocado peel extracts showed a direct relationship between antioxidant activities and polyphenol/flavonoid content, where the extracts with the highest polyphenols/flavonoids showed the best antioxidant activities [22]. Similarly, a study on *Centella asiatica* extracts found that the water extract, with its higher TFC (30.09 mg QE/g), showed stronger antioxidant activity in an ABTS assay when compared to the ethanol (EtOH) extract (23.03 mg QE/g) [23].

In conclusion, our results consistently show a positive correlation between higher TPC and TFC in FG extracts and their antioxidant activities, as measured by various assays such as DPPH and ABTS. This is in line with established studies in the field. However, in a study of the antioxidant activity in *Rosa damascena* Mill. from the same Rosaceae family [24], the highest antioxidant activity was observed in the early developmental stages of the plant (bud stage) and decreased with flowering and senescence. The authors speculated that this pattern might be due to the changing roles of antioxidants and antioxidant enzymes during flower development, which require higher activity in the early stages to manage the oxidative stress associated with rapid growth and development, thus making them contrary to our results.

### 2.3. Anti-Inflammatory Activities

The anti-inflammatory activities of FG were assessed by evaluating its ability to inhibit the production of inflammation-associated biomarkers, such as NO and IL-6, in LPS -stimulated RAW 264.7 macrophage cell supernatants using the Griess assay and enzyme-linked immunosorbent assay (ELISA), respectively. RAW 264.7 cells are a murine macrophage cell line with macrophage-like properties capable of producing cytokines and NO [25]. The cytotoxic effects of different FG extracts were first evaluated using the soluble 3-(4,5-dimethylthiazol-2-yl)-2,5-diphenyltetrazolium bromide (MTT) assay to establish safe concentrations for subsequent in vitro experiments. According to ISO 10993-5 standards for in vitro cytotoxicity, a cell viability above 80% is considered non-cytotoxic [26]. 

**Figure 1:**
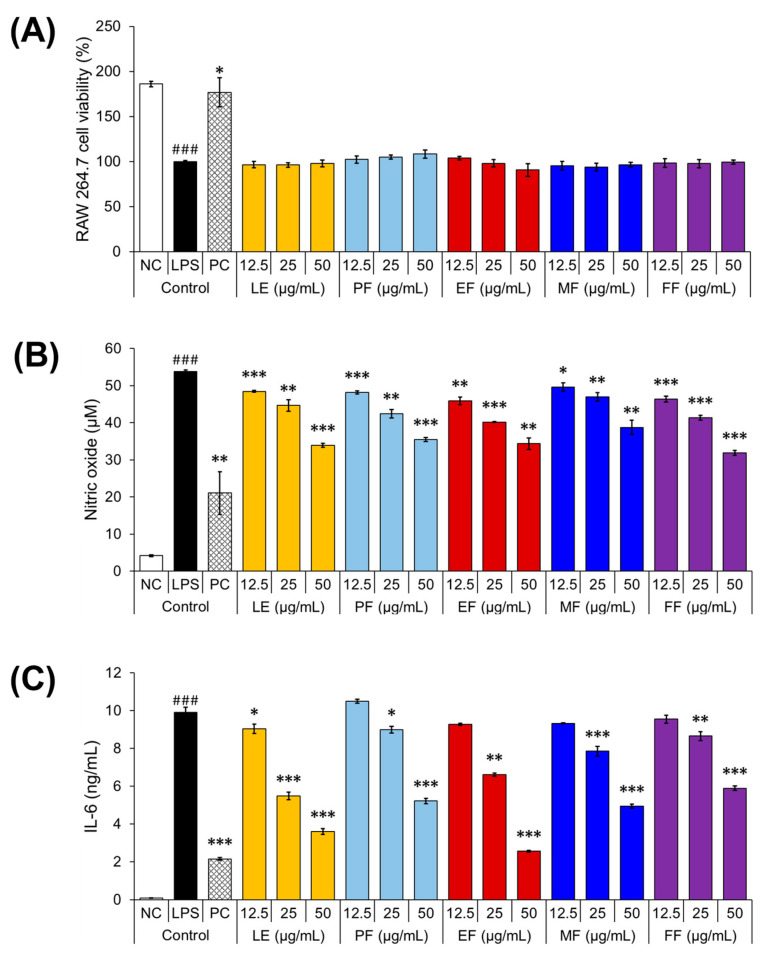
Cytotoxic effects of the FG extracts on the LPS-stimulated RAW 264.7 cells (**A**), and the anti-inflammatory activities of FG extracts from different stages (**B**,**C**). The figure shows the effect of the FG extracts on the production of NO and IL-6 in the LPS-stimulated RAW 264.7 cells. The superscripts indicate different levels of significant difference between the LPS and each FG extract. *, *p* < 0.05; **, *p* < 0.01; and ^###^ or ***, *p* < 0.001. FG, *F. glaberrima.* NC, negative control (without LPS stimulation). PC, positive control (dexamethasone, with LPS stimulation). LPS, lipopolysaccharides. LE, 2 months after leaf emergence. PF, pre-flowering. EF, early flowering. MF, mid-flowering. FF, full-flowering.

Figure 1A shows that none of the FG extracts, which were tested at concentrations ranging from 12.5–50 μg/mL, exhibited significant cytotoxic effects on the LPS-stimulated RAW 264.7 cells (LE, 96.2–97.8%; PF, 102.3–108.2%; EF, 90.6–103.8%; MF, 93.7–96.6%; and FF, 96.4–99.4%) compared to the LPS-treated control (100%). As expected, the negative control showed the highest cell viability, whereas the positive control, using the anti-inflammatory drug dexamethasone, showed a significant increase in cell viability compared to the LPS-stimulated control.

LPS treatment triggers the release of pro-inflammatory cytokines and activates the secondary inflammatory cascades involving other cytokines, lipid mediators, and the regulatory and effector molecule NO [27,28,29]. All FG extracts showed a significant concentration-dependent decrease in NO compared to the LPS-treated control (Figure 1B). Specifically, compared to the LPS-stimulated control, LE showed 48.5–18.5 μM and 10.7–71.3% inhibition; PF showed 48.2–26.1 μM and 11.2–55.8% inhibition; EF showed 46.4–18.4 μM and 15.8–71.4% inhibition; MF showed 49.6–26.0 μM and 8.5–56.1% inhibition; and FF showed 46.4–21.7 μM and 15–64.8% inhibition. The highest level of inhibition was observed at the maximum FG dose of 50 μg/mL. In addition, there was a dose-dependent inhibition of IL-6 production at the equivalent concentrations above (Figure 1C). IL-6 is synthesized at the onset of inflammation and has pleiotropic effects [30]. LE showed the significant inhibition of IL-6 at all tested concentrations (9.0–3.6 ng/mL; 9–64.3% inhibition), whereas PF (10.5–5.2 ng/mL; 5.6–47.8% inhibition), EF (9.3–2.6 ng/mL; 6.4–74.9% inhibition), MF (9.3–4.9 ng/mL; 5.9–50.6% inhibition), and FF (9.5–5.9 ng/mL; 3.8–41.0% inhibition) showed a significant reduction in IL-6 levels only at 25–50 μg/mL. These results demonstrate the ability of all FG extracts at different growth stages to counteract the production of pro-inflammatory biomarkers in LPS-stimulated RAW 264.7 macrophage cells.

### 2.4. Anti-Atopic Activities

**Figure 2:**
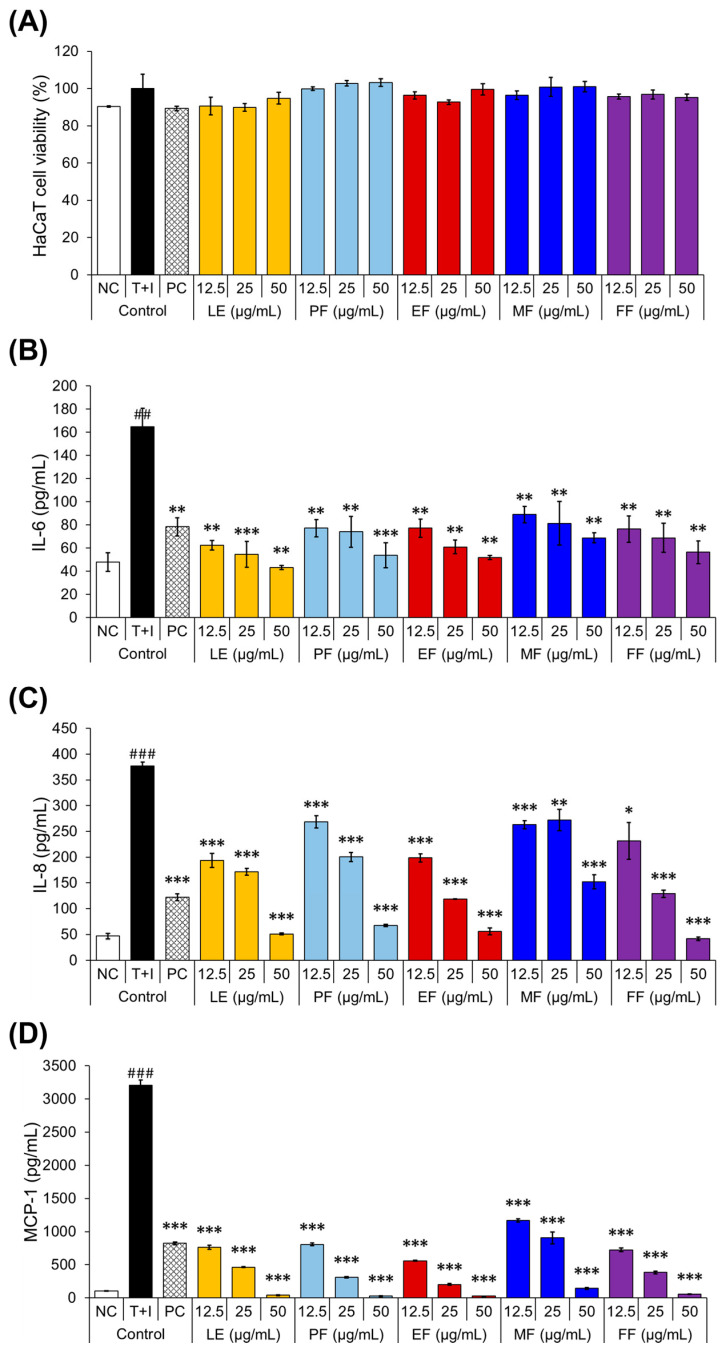
Cytotoxic effects of the FG extracts on the T+I-stimulated HaCaT cells (**A**), and the anti-atopic activities of FG (**B**–**D**). The figure shows the effect of the FG extracts on the production of IL-6, IL-8, and MCP-1 in the T+I-stimulated HaCaT cells. * and ^#^ superscripts indicate different levels of significant difference between the T+I-stimulated cells and each FG extract and T+I-stimulated cells and NC, respectively. *, *p* < 0.05; ^##^ or **, *p* < 0.01; and ^###^ or ***, *p* < 0.001. FG, *F. glaberrima.* NC, negative control (without T+I stimulation). PC, positive control (dexamethasone, with T+I stimulation). IFN-γ, interferon-gamma. TNF-α, tumor necrosis factor-alpha. T+I, TNF-α + IFN-γ. LE, 2 months after leaf emergence. PF, pre-flowering. EF, early flowering. MF, mid-flowering. FF, full-flowering.

The suppression of cytokine and chemokine production serves as a valuable biomarker of anti-atopy activity. Therefore, the effects of FG extracts on skin inflammation were assessed using HaCaT cells as keratinocytes play a critical role in the complex cellular interactions during inflammatory responses in the skin [31]. Prior to the subsequent experiments, cell viability was assessed using similar concentrations (12.5–50 μg/mL) that were used in the anti-inflammatory experiment. The MTT assay was used to compare the cytotoxicity in the HaCaT cells treated with FG. Figure 2A shows that none of the FG extracts exhibited cytotoxicity as the cell viability was not significantly different compared to the TNF-α + IFN-γ (T+I) stimulated control. Notably, the viability of FG extracts, which ranged from 95.3 to 104.6%, was considered non-cytotoxic and has shown consistent results with RAW 264.7 cells.

The cytokine and chemokine secretion in FG-treated T+I-stimulated HaCaT cells was assessed by ELISA. The cytokines (IL-6, IL-8) and chemokine (MCP-1) levels were significantly increased after T+I stimulation, as shown in Figure 2B–D, whereas all FG extracts at different concentrations resulted in a significant concentration-dependent reduction in the production of these inflammatory mediators. Specifically, the IL-6 levels were significantly inhibited by LE (62.6–43.2 pg/mL; 87.6–104.2% inhibition), PF (77.2–53.8 pg/mL; 75–95.1% inhibition), EF (77.2–51. 8 pg/mL; 75–96.8% inhibition), MF (89.0–68.8 pg/mL; 64.9–82.2% inhibition), and FF (76.5–56.4 pg/mL; 75.7–92.8% inhibition) stage extracts compared to the T+I-stimulated group (164.9 pg/mL).

A dose-dependent decrease in IL-8 levels was also observed with LE (193.5–50.7 pg/mL; 55.5–98.7% inhibition), PF (268.4–67.3 pg/mL; 32.8–93.7% inhibition), EF (198.6–55.8 pg/mL; 54.0–97.2% inhibition), MF (262.8–151.9 pg/mL; 34.5–68.1% inhibition), and FF (231.6–28.5 pg/mL; 43.9–101.4% inhibition) stage extracts compared to that in the T+I-stimulated group (376.7 pg/mL). FG extracts also significantly reduced MCP-1 levels in the T+I-stimulated HaCaT cells. The LE (763.1–40.3 pg/mL; 78.8–102.1% inhibition), PF (807.1–27.7 pg/mL; 77.4–102.5% inhibition), EF (559.1–26.1 pg/mL; 85.4–102.6% inhibition), MF (1169.4–143.0 pg/mL; 65.7–98.8% inhibition), and FF (723.2–55.5 pg/mL; 80.1–101.6% inhibition) stages significantly inhibited MCP-1 production compared to the T+I-stimulated group (376.7 pg/mL). These results indicate that FG treatments can inhibit the expression of cytokines and chemokines induced by the T+I in HaCaT cells, highlighting their potential as anti-atopic agents for skin inflammation.

### 2.5. Characterization of Phytochemical Content by HPLC Analysis

**Figure 3:**
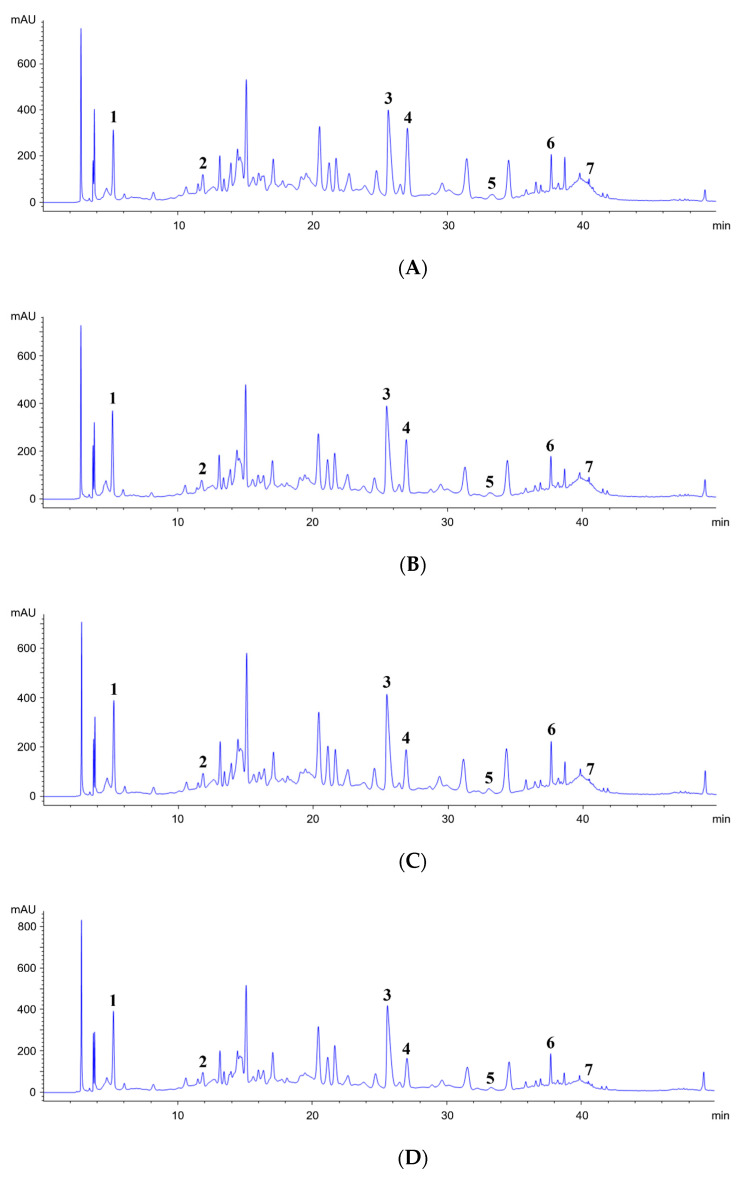

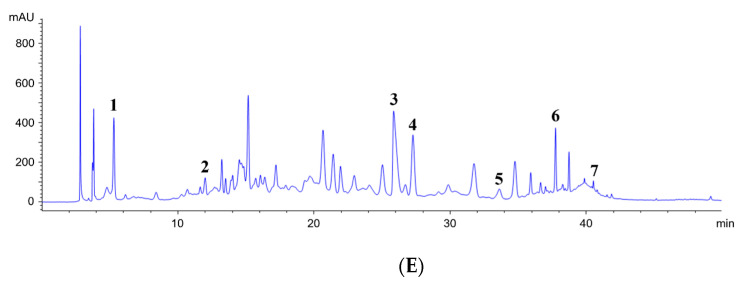
HPLC chromatograms of the LE (**A**), PF (**B**), EF (**C**), MF (**D**), and FF (**E**) stages (**1**: gallic acid, **2**: (+)-catechin, **3**: hyperin, **4**: miquelianin, **5**: astragalin, **6**: afzelin, and **7**: quercetin). LE, 2 months after leaf emergence; PF, pre-flowering; EF, early flowering; MF, mid-flowering; and FF, full-flowering.

HPLC analysis was used to investigate the phytochemical content of the FG samples at different growth stages. The HPLC chromatogram (Figure 3) showed good separations, and Table 2 shows the retention time and other parameters for quantification. The FG samples were harvested at five different flowering stages. Among all the flowering stages analyzed (Table 3), the FF stage exhibited the highest total phytochemical content (43.33 mg/g).

Further analysis showed that the FF stage had the highest levels of all the individual phytochemicals analyzed, from gallic acid to quercetin. Although external environmental factors such as growth conditions may have played a role [32,33,34,35,36], the significantly higher content of all the phytochemicals in the FF stage suggested that the developmental stage of the plant is likely to have the most significant influence on phytochemical variation. This finding is consistent with observations of the higher flavonoid and phytochemical content in FF stage plants, such as the *Achillea collina* and *Fagopyrum* species [34,37]. In addition, research on American cranberries suggests that the highest concentration of flavonoid compounds is present in the flower ovary, which decreases during fruit development followed by a subsequent increase during ripening [38]. Similarly, in several plants, the levels of secondary metabolites tend to peak in flowers and decrease during later stages of seed development [34,39,40], a trend that can be applied to interpret the results of this experiment. Thus, further investigation of the correlation between the genes involved in different biosynthetic pathways in FG and the variation in phytochemical content with the growth stage may provide insights into the origins of these differences.

## 3. Materials and Methods

### 3.1. Plant Materials

The seeds of FG collected in June 2020 in Yeoncheon, Korea were planted in an open field and used in the experiment [7]. FG specimens were cultivated in Eumseong, Korea, in June 2021. They were divided into five taxa according to the growth stages of FG and were identified by Dr. C. G. Park, National Institute of Horticultural and Herbal Science, Korea. Voucher specimens were deposited at the herbarium of the Department of Plant Science and Technology, Chung-Ang University, Anseong, Korea. For this study, only the leaves of FG were used because they are the primary site of secondary metabolite production and can be sampled non-destructively throughout the growing season.

### 3.2. Instruments, Materials, and Reagents

**Figure 4:**
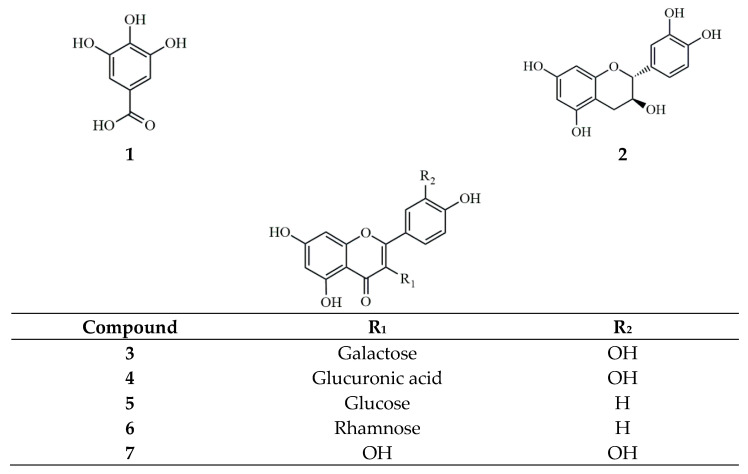
Chemical structures of compounds **1**–**7** from *F. glaberrima* (**1**: gallic acid, **2**: (+)-catechin, **3**: hyperin, **4**: miquelianin, **5**: astragalin, **6**: afzelin, and **7**: quercetin).

Chromatographic analysis was performed using an HPLC system (Agilent 1260 Infinity II Quat Pump, Santa Clara, CA, USA), consisting of an autosampler, pump, and diode array detector (DAD WR detector, Santa Clara, CA, USA). HPLC-grade solvents, including water, methanol (MeOH), and acetonitrile (ACN), were purchased from J. T. Baker (Phillipsburg, PA, USA). Trifluoroacetic acid (HPLC grade) was purchased from Thermo Scientific (Waltham, MA, USA). All compounds (gallic acid, (+)-catechin, hyperin, miquelianin, astragalin, afzelin, and quercetin) were obtained from the Natural Product Institute of Science and Technology (www.nist.re.kr; accessed on 27 April 2022), Anseong, Korea (Figure 4). The ^1^H–NMR and ^13^C–NMR spectra were recorded with an AVANCE III HD 500 NMR spectrometer (Bruker, Hanau, Germany). The spectrometer operated at 500 MHz for ^1^H-NMR and 125 MHz for ^13^C-NMR with a 5 mm BBFO (plus) probehead. Tetramethylsilane was used as the internal standard. For in vitro experiments, DPPH and ABTS solutions were purchased from Sigma (St. Louis, MO, USA). Dulbecco’s modified Eagle’s medium (DMEM; Cat. No. D6429-500ML), MTT (298-93-1), dexamethasone (CAS No. 50-02-2), Griess reagent (CAS No. 1465-25-4), and LPS (L4391) were purchased from Sigma Aldrich (St. Louis, MO, USA). Fetal bovine serum (FBS; Cat. No. 35-015-CV) and the antibiotics penicillin and streptomycin (P/S; Cat. No. 30-002-CI) were provided by Corning Life Sciences (Manassas, VA, USA). Dimethyl sulfoxide (DMSO; CAS No. 67-68-5) was obtained from Duchefa Biochemie (Haarlem, The Netherlands). Recombinant human interferon-gamma (IFN-γ) protein (Cat. No. 285-IF-100) and recombinant human TNF-α protein (Cat. No. 210-TA-100) (T+I) were purchased from R&D Biosystem (Minneapolis, MN, USA). The ELISA kits for IL-6 (IL-6; Cat. No. 555220), IL-8 (Cat. No. 555244), and MCP-1 (Cat. No. 555260) were purchased from BD Biosciences (San Diego, CA, USA).

### 3.3. Extraction and Isolation

FG shade-dried leaves (2.1 kg) were extracted with EtOH under reflux at 80 °C, and this was conducted five times for 3 h using 20 times the amount of EtOH relative to the sample. The EtOH extract (346.4 g) was evaporated, suspended in distilled water, and partitioned sequentially with *n*-hexane (11.1 g), chloroform (2.3 g), ethyl acetate (33.9 g), and *n*-butanol (21.0 g). Part of the ethyl acetate and *n*-butanol fractions (20 and 15 g, respectively) were subjected to open-column chromatography. The mobile phase used for the isolation was chloroform-MeOH, with gradually increasing proportions of MeOH. After the primary separation, ODS C18 and Sephadex LH-20 were used for further separation and purification. Open-column chromatography was performed on a silica-gel column (60–200 mesh, Zeochem, Switzerland), LiChroprep RP-18, and Sephadex LH-20 (Sigma Aldrich, St. Louis, MO, USA). The MeOH recrystallization using solubility differences was used for the final isolation of the phytochemicals. This approach led to the isolation of gallic acid, (+)-catechin, hyperin, miquelianin, astragalin, afzelin, and quercetin from the FG extract (Figure 4). The structures of the isolated compounds were confirmed by ^1^H- and ^13^C-NMR analysis.

### 3.4. Analysis of the TPC and TFC

The TPC and TFC in the FG extracts at different growth stages were quantified according to established methods [41]. For the TPC, 60 μL of 2N Folin-Ciocalteu phenol reagent (Sigma-Aldrich, St. Louis, MO, USA) was added to 60 μL of extract. The mixture was allowed to react with 60 μL of 15% Na_2_CO_3_ (Daejung Chemicals, Siheung, Republic of Korea) for 30 min. The TFC was measured by adding 100 μL of 2% AlCl_3_ to 100 μL of the extract and incubating the mixture for 10 min. Absorbance measurements for the TPC and TFC were performed at 760 and 430 nm, respectively, using a microplate reader. Gallic acid and quercetin were used as standards to generate the calibration curves. All experimental procedures were performed at room temperature.

### 3.5. DPPH Radical Scavenging Activity

The DPPH method is widely used to measure the antioxidant activity of natural product extracts. In this experiment, the antioxidant activity of FG extracts was measured using the method described by Doan et al. [42]. First, 200 μL of 0.2 mM DPPH was dissolved in 95% EtOH, and 10 μL of each FG extract were added to a microtube, vortexed, and reacted in the dark for 30 min. The concentrations of the remaining radicals were measured using a microplate reader at 514 nm. Ascorbic acid (Sigma, USA) was used as a positive control. The scavenging ability (IC_50_) of the extract against DPPH was expressed as the concentration required to reduce 50% of the the absorbance of the control using only the solvent:

DPPH radical-scavenging activity (%) = (Blank O.D − Sample O.D)/Blank O.D × 100.

### 3.6. ABTS Radical Scavenging Activity

The ABTS radical scavenging activity of the FG extracts was measured by modifying a previously reported method [43]. It involved dissolving 7.4 mM of ABTS and potassium persulfate (2.6 mM) in distilled water, which was added in a 1:1 ratio. This was further diluted with distilled water to an absorbance of 1.00 ± 0.04. After allowing the mixture to stand in the dark for 24 h, 10 μL of each sample, which were prepared by concentration in 200 μL of radical stock solution, was added. After allowing the mixture to stand for 30 min, the concentrations of the remaining radicals were measured using a microplate reader at 734 nm. Ascorbic acid was used as a positive control. The IC_50_ of the extract against ABTS was expressed as the concentration required to reduce the absorbance of the control by 50% using only the solvent:

ABTS radical-scavenging activity (%) = (Blank O.D − Sample O.D)/Blank O.D × 100.

### 3.7. Evaluation of Anti-Inflammatory Activity

The RAW 264. 7 cells, obtained from the Korean Cell Line Bank (KCLB; Seoul, South Korea), were used to evaluate the anti-inflammatory activities of the FG samples. The cells were seeded at a density of 6.0 × 10^5^ cells/mL in a 96-well culture plate using DMEM supplemented with 10% FBS and 1% P/S, and they were then maintained in a humidified incubator at 37 °C with 5% CO_2_. The seeded cells were first stabilized for 24 h. The medium was then replaced with fresh DMEM and treated with FG extracts at concentrations ranging from 12.5–50 μg/mL. The positive and negative controls used were 1% DMSO/serum-free medium (SFM)-DMEM and dexamethasone (50 μg/mL), respectively. Cells were initially incubated for 30 min before the addition of LPS (1 μg/mL) to induce inflammation. After 24 h, the potential cell cytotoxicity was assessed using a commercially available MTT assay. The cell supernatant was collected to assess NO production using the Griess assay and cytokine (IL-6) levels, which werequantified using ELISA.

### 3.8. Evaluation of Anti-Atopy Activity

The anti-atopic effects were assessed using a human keratinocyte cell line (HaCaT cells) obtained from the CLS Cell Line Service (Eppelheim, Heidelberg, Germany). Cells were seeded at 2.0 × 10^5^ cells/mL in a 96-well culture plate using DMEM supplemented with 10% FBS and 1% P/S. The cells were first stabilized for 24 h and then incubated with SFM-DMEM for another 24 h. After incubation, the cells were treated similarly to the RAW 264.7 cells; however, T+I (10 ng/mL) was used instead of LPS to induce inflammation. Additionally, dexamethasone as a positive control was added at a concentration of 20 μg/mL. After further incubation for 24 h, the cell viability was assessed by MTT assay, and the levels of cytokines (IL-6 and IL-8) and chemokine (MCP-1) in the collected cell supernatants were quantified by ELISA.

### 3.9. Cell Viability, Griess Assay, and ELISA

The MTT assay was used to evaluate the viability and cytotoxicity of the FG extracts on the RAW 264.7 and HaCaT cell lines. After a 24 h incubation, the cells were mixed with 0.1 mg/mL of MTT/SFM-DMEM (100 μL) and incubated for 30 min. The medium was then removed and 100 μL of DMSO was added. The optical density was measured at 550 nm using an Epoch spectrophotometer (BioTek Instruments, Inc., Winosky, VT, USA). An absorbance value lower than the LPS and the T+I-stimulated controls indicated a reduction in the rate of viable cells.

The level of NO production was determined using the Griess assay, which detects and quantifies NO through its stable product—nitrite. The RAW 264.7 cell supernatant was mixed with 50 μL of Griess reagent (Sigma-Aldrich Co., St. Louis, MO, USA) and incubated in a dark room at 20–25 °C for 15 min. The optical density was then measured at 540 nm.

The inhibition of cytokine and chemokine production mediated by FG extracts was evaluated using cell supernatants from LPS-stimulated RAW 264.7 and T+I-induced HaCaT cells after the respective incubation periods. The cytokine and chemokine levels were measured using specific ELISA kits according to the manufacturer’s protocols. Details of the ELISA kits are provided in the instruments, materials, and reagents section.

### 3.10. Preparation of the Sample and Standard Solutions for HPLC

Shade-dried FG leaves (10 g) from different growth stages were extracted three times for 3 h each using 20 times their weight in EtOH under a reflux at 80 °C. The extracts were evaporated in vacuo. The EtOH extract was dissolved in 80% MeOH (30 mg/mL) and filtered through a syringe filter (PVDF, 0.22 μm). The stock solutions of the standards were prepared by dissolution in 80% MeOH (0.5 mg/mL). Working solutions were prepared by diluting the stock solutions to the desired concentrations for the standard calibration curves.

### 3.11. HPLC/UV Conditions

Comparative analysis was performed on a reversed-phase HPLC system using an INNO C18 column (250 mm × 4.6 mm, 5 μm) at ambient temperature. The injection volume was 10 μL, and the system was monitored at 270 nm. The flow rate was adjusted to 1.0 mL/min. The mobile phase consisted of 0.1% trifluoroacetic acid in water (A) and ACN (B), and the gradient elution was achieved as follows: 90% A at 0 to 5 min; 82% A from 5 to 10 min; decreased to 78% A from 10 to 30 min; decreased to 65% A from 30 to 35 min; decreased to 0% A from 35 to 45 min and maintained until 50 min; and then increased to 90% A from 50 to 55 min and maintained until 65 min.

### 3.12. Calibration Curve

A calibration curve was constructed by plotting the concentrations of the standard solutions against their respective peak areas. The linearity of the calibration curve was determined from the correlation coefficient (*r*^2^). The standard concentrations in the samples were then calculated from the calibration curve. The calibration functions were determined based on the peak area (Y), concentration (X, mg/mL), and mean ± standard deviation (SD) (n = 3).

### 3.13. Statistical Analysis

All experiments were performed in triplicate, and the results are expressed as the mean ± SD for the in vitro studies. Student’s *t*-test was used for group comparisons, with * *p* < 0.05, ** *p* < 0.01, and *** *p* < 0.001 indicating statistical significance. An analysis of variance (ANOVA) test and Tukey’s test were performed at the 95% confidence level (*p* < 0.05) using Minitab statistical software (Minitab Version 16, State College, PA, USA) to determine the significant differences in the TPC, TFC, antioxidant activity, and HPLC data.

## 4. Conclusions

A comprehensive analysis of FG extracts at different growth stages revealed a significant correlation between the developmental stage and the accumulation of bioactive compounds such as polyphenols and flavonoids, which, in turn, influenced the antioxidant, anti-inflammatory, and anti-atopic activities of the plant. The highest concentrations of TPC and TFC were observed during the FF phase, which is consistent with the established research that the flowering phase is critical for peak accumulation of these phytochemicals. This phase also showed the strongest antioxidant activity, thus confirming a positive correlation between high phytochemical content and antioxidant efficacy. Furthermore, the FG extracts exhibited significant anti-inflammatory effects, with the LE and FF stages showing the greatest inhibition of inflammatory markers such as the NO and IL-6 in the LPS-stimulated RAW 264.7 macrophage cells. These extracts also showed promising anti-atopic properties by significantly reducing the levels of inflammatory cytokines (IL-6 and IL-8) and chemokine (MCP-1) in T+I-stimulated HaCaT cells. Additionally, the non-cytotoxic nature of these extracts at various concentrations supports their potential therapeutic application. Finally, the HPLC analysis highlighted the FF stage as the peak period for the highest content of individual phytochemicals (gallic acid, (+)-catechin, hyperin, miquelianin, astragalin, afzelin, and quercetin), thus reinforcing the notion that the flowering stage is critical for maximum phytochemical yield. This highlights the importance of the FF stage in FG in maximizing the extraction of bioactive compounds, and it also suggests the potential for further research on the genetic and biosynthetic pathways responsible for these variations. Understanding these mechanisms could facilitate the development of FG-based applications in pharmaceutical and biotechnological fields.

## Figures and Tables

**Table 1:** The TPC, TFC, ABTS, and DPPH activities at different stages of *F. glaberrima*.

Samples	TPC (mg GAE/mL)	TFC (mg QE/mL)	IC_50_ (mg/mL)	
			ABTS	DPPH
LE	12.75 ± 1.91 ^ab^	7.49 ± 0.59 ^a^	0.23 ± 0.01 ^c^	0.69 ± 0.03 ^a^
PF	9.06 ± 1.50 ^bc^	5.31 ± 0.29 ^b^	0.27 ± 0.01 ^ab^	0.67 ± 0.06 ^ab^
EF	7.01 ± 0.91 ^c^	5.97 ± 0.13 ^ab^	0.25 ± 0.00 ^bc^	0.65 ± 0.06 ^ab^
MF	5.53 ± 0.90 ^c^	5.37 ± 0.23 ^b^	0.27 ± 0.00 ^a^	0.72 ± 0.07 ^a^
FF	14.75 ± 2.69 ^a^	7.75 ± 1.31 ^a^	0.16 ± 0.01 ^d^	0.55 ± 0.02 ^b^
Ascorbic acid			0.10 ± 0.00	0.08 ± 0.00

^a–d^ Different letters within the same column indicate significant statistical differences (*p* < 0.05) according to Tukey’s test. LE, 2 months after leaf emergence; PF, pre-flowering; EF, early flowering; MF, mid-flowering; and FF, full-flowering.

**Table 2:** The calibration curves of the phytochemicals of *F. glaberrima*.

Compound	t_R_	Linear Range (µg/mL)	LOD (µg/mL)	LOQ (µg/mL)	Calibration Equation	Correlation Factor, *r*^2^
Gallic acid (1)	5.01	0–500	0.33	0.98	Y = 32.134X + 169.17	1.0000
(+)-Catechin (2)	11.9	0–500	0.48	1.45	Y = 3.3541X + 31.146	0.9996
Hyperin (3)	25.9	0–500	0.39	1.19	Y = 15.666X + 79.491	0.9999
Miquelianin (4)	27.1	0–500	0.23	0.69	Y = 19.653X + 81.667	1.0000
Astragalin (5)	33.2	0–500	0.31	0.95	Y = 16.769X + 65.244	0.9998
Afzelin (6)	37.9	0–500	0.81	2.41	Y = 14.891X + 137.51	0.9995
Quercetin (7)	40.3	0–50	0.54	1.65	Y = 23.209X + 19.113	0.9995

t_R_ = retention time; LOD = limits of detection, LOQ = limits of quantification; Y = peak area, X = concentration of the standard (µg/mL); and *r*^2^ = correlation coefficient for the five data points in the calibration curve.

**Table 3:** Content of Compounds **1**–**7** in *F. glaberrima*.

Compound	Content (mg/g ext.)				
	LE	PF	EF	MF	FF
Gallic acid (1)	1.98 ± 0.02 ^e^	2.47 ± 0.02 ^d^	2.73 ± 0.01 ^b^	2.59 ± 0.01 ^c^	2.84 ± 0.02 ^a^
(+)-Catechin (2)	9.90 ± 0.02 ^b^	7.91 ± 0.17 ^c^	7.57 ± 0.05 ^d^	5.12 ± 0.05 ^e^	10.30 ± 0.07 ^a^
Hyperin (3)	12.76 ± 0.03 ^e^	13.12 ± 0.07 ^d^	13.77 ± 0.03 ^c^	14.45 ± 0.02 ^b^	16.21 ± 0.03 ^a^
Miquelianin (4)	7.11 ± 0.03 ^b^	5.42 ± 0.03 ^c^	3.95 ± 0.06 ^d^	3.41 ± 0.01 ^e^	7.62 ± 0.01 ^a^
Astragalin (5)	0.94 ± 0.03 ^b^	0.71 ± 0.02 ^d^	0.84 ± 0.01 ^c^	0.60 ± 0.01 ^e^	2.12 ± 0.02 ^a^
Afzelin (6)	1.92 ± 0.01 ^c^	1.65 ± 0.02 ^e^	2.27 ± 0.02 ^b^	1.77 ± 0.01 ^d^	4.06 ± 0.02 ^a^
Quercetin (7)	0.11 ± 0.00 ^b^	0.10 ± 0.00 ^c^	tr	tr	0.18 ± 0.00 ^a^
Total content	34.72	31.38	31.13	24.53	43.33

^a–e^ Different letters within the same row indicate significant statistical differences (*p* < 0.05) according to Tukey’s test; tr = trace amount; LE, 2 months after leaf emergence; PF, pre-flowering; EF, early flowering; MF, mid-flowering; and FF, full-flowering.

## Data Availability

The original contributions presented in the study are included in the article, further inquiries can be directed to the corresponding author.
